# Obesity and Male Reproduction; Placing the Western Diet in Context

**DOI:** 10.3389/fendo.2021.622292

**Published:** 2021-03-11

**Authors:** Taylor Pini, David Raubenheimer, Stephen J. Simpson, Angela J. Crean

**Affiliations:** Charles Perkins Centre, School of Life and Environmental Sciences, The University of Sydney, Sydney, NSW, Australia

**Keywords:** obesity, male fertility, reproduction, diet, high fat, nutritional geometry

## Abstract

There is mounting evidence that obesity has negative repercussions for reproductive physiology in males. Much of this evidence has accumulated from rodent studies employing diets high in fat and sugar (“high fat” or “western” diets). While excessive fats and carbohydrates have long been considered major determinants of diet induced obesity, a growing body of research suggests that the relationships between diet composition and obesity are more complex than originally thought, involving interactions between dietary macronutrients. However, rodent dietary models have yet to evolve to capture this, instead relying heavily on elevated levels of a single macronutrient. While this approach has highlighted important effects of obesity on male reproduction, it does not allow for interpretation of the complex, interacting effects of dietary protein, carbohydrate and fat. Further, the single nutrient approach limits the ability to draw conclusions about which diets best support reproductive function. Nutritional Geometry offers an alternative approach, assessing outcomes of interest over an extended range of dietary macronutrient compositions. This review explores the practical application of Nutritional Geometry to study the effects of dietary macronutrient balance on male reproduction, including experimental considerations specific to studies of diet and reproductive physiology. Finally, this review discusses the promising use of Nutritional Geometry in the development of evidence-based pre-conception nutritional guidance for men.

## Introduction

**Figure 1:**
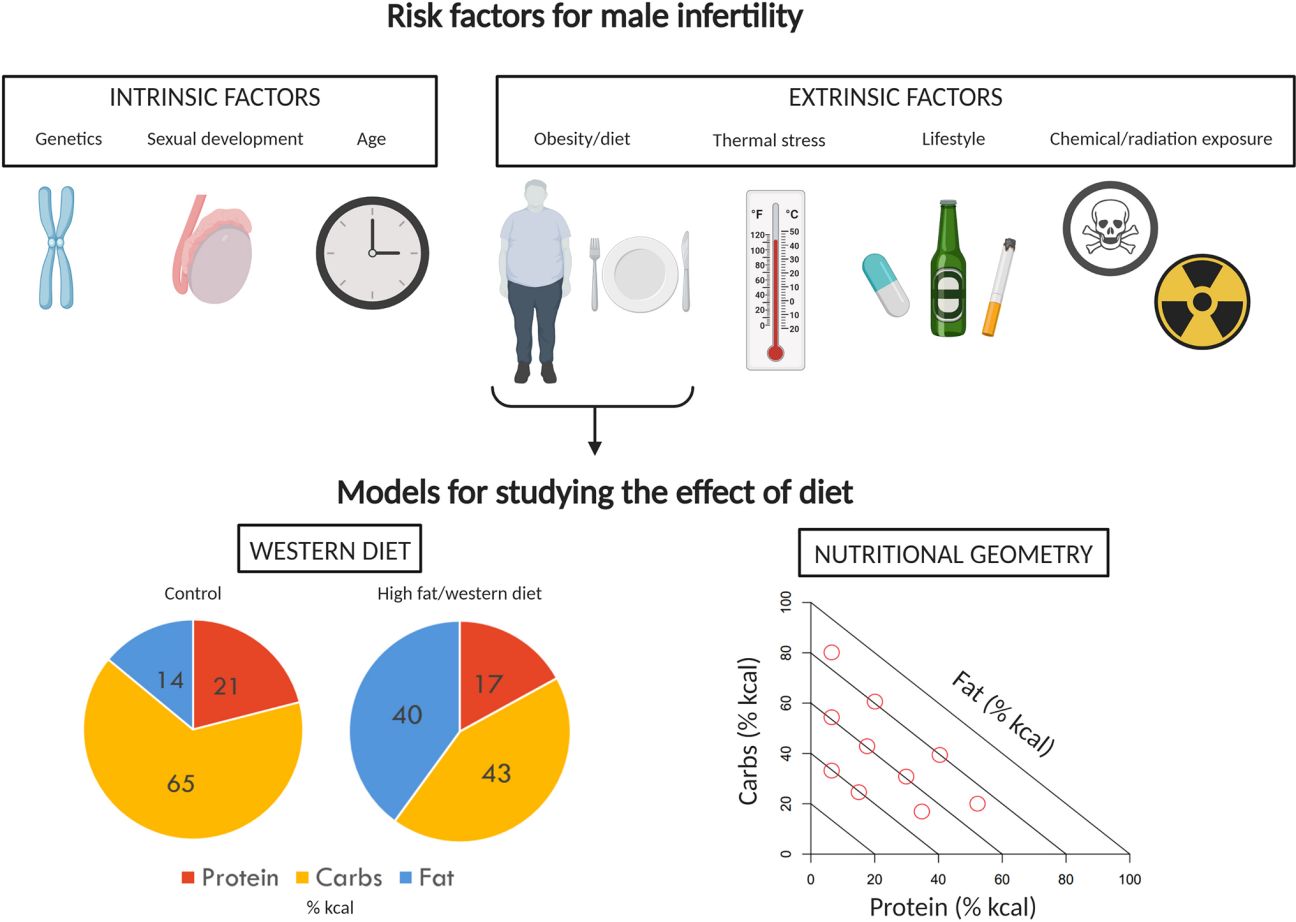
Factors impacting male fertility and models to study effects of diet. A variety of intrinsic and extrinsic factors can contribute to poor reproductive performance and infertility in men. Of the extrinsic factors, the impact of diet and obesity on male reproduction has received a significant amount of attention. The effects of diet and obesity on male reproduction are typically investigated in rodents using the classic “western diet” approach, comparing control and treatment diet outcomes. Here, we instead propose the use of Nutritional Geometry, employing a range of diets which systematically differ in their proportions of protein, carbohydrates, and fat. Image created with BioRender.com.

Obesity affects millions of people globally. Men of reproductive age (18–64 years) are no exception, with averages of 37.8% (1), 31.4% (2), and 29.9% (3) classified as obese (body mass index ≥ 30 kg/m^2^) in the United States, Australia, and the United Kingdom, respectively. Because of its epidemiological prevalence, the impacts of obesity have been studied in the context of many biological processes, including reproduction. While much of the literature has focused on female reproduction, a growing body of evidence suggests that obesity and associated metabolic dysfunction can alter spermatozoa on a molecular level (4, 5), negatively affect sperm function (6–8), alter circulating levels of reproductive hormones (9), cause male sub-fertility (10, 11) and impart epigenetic changes to spermatozoa which ultimately decrease offspring metabolic health (12) and reproductive potential (13). In addition, a wide range of intrinsic [e.g., DAZ deletion (14), age (15)] and extrinsic [e.g., radiation exposure (16), tobacco use (17)] factors can contribute to male infertility, and may interact with or compound the effects of obesity on male reproduction (**Figure 1**). While male obesity is generally recognized as an important concern in the context of reproductive medicine (18–20), some studies question the effects of obesity on semen parameters and male fertility (21–25). These inconsistencies and the seriousness of the potential consequences of obesity on male fertility necessitate continued research efforts in this field.

The root cause of obesity is a topic which has been debated for decades. While there is a growing number of risk factors associated with obesity, including sleep, genetic background, and physical activity, diet is the most significant contributor (26). Many have identified lipid as the major dietary determinant (27, 28), but this has been refuted by others who consider carbohydrate to be the major culprit (29–31). In contrast to these single-nutrient explanations, there is mounting evidence that obesity may instead be driven by an altered macronutrient balance in the diet, rather than by high dietary fat or carbohydrate alone (32–34). Despite this, animal studies employing purified diets with elevated concentrations of fat (typically referred to as “high fat” or “western” diets) remain a staple of research investigating diet induced obesity (35). As the understanding of what constitutes an obesogenic diet changes, there is a need to rethink the dietary models which are used to study obesity and its downstream consequences for such factors as reproduction.

In this review, we summarise the limitations of the traditional western diet approach and introduce Nutritional Geometry as a powerful framework for studying the relationships between diet, obesity, and male reproduction. We also highlight important experimental design considerations unique to studying male reproductive physiology. Finally, we pose potential applications for Nutritional Geometry in the context of male reproduction, including a path toward the development of pre-conception nutritional guidelines.

## The Current Approach to Studying Obesity and Male Reproduction

Evidence from animal studies indicates that obesity and associated metabolic disease are deleterious for male reproduction (**Table 1**). A variety of processes are reportedly impacted by obesity, including testosterone production (7), testicular gene expression (36–38), production of reactive oxygen species (6, 8), and maintenance of the blood-testis barrier (7). These studies compare the effects of diets which contain normal (10%–18% of total kcal) or high (40%–60% of total kcal) amounts of fat, with some also incorporating elevated levels of sucrose. Rodent diets high in the proportion of fat are excellent tools to create an obese phenotype, resulting in significantly larger adipose tissue depots (6) and a higher overall percentage body fat compared to lean mass (37, 42). These high-fat diets also often capture metabolic sequelae, including elevated serum cholesterol, triglycerides, glucose, insulin, and leptin (6, 37, 42), though this is not always the case (43–45). While it would be easy to conclude from these studies that avoiding a high-fat diet will safeguard reproductive potential, the reality is more complicated.

**Table 1:** Rodent studies employing a traditional high fat/western diet approach to study the impact of obesity on male reproduction.

Reference	Diet	Diet type	Species	Age at start	Treatment length	kcal/g	% protein	% carb	% fat	P:C	P:F	Unique ingredients	Outcomes			
													Testis	Epididymis/Accessory sex glands	Sperm	Mating/offspring
(7)	MD12031 10% fat, Mediscience Ltd	Grain-based	Mouse (C57BL/6)	5 wk	10 wk	–	20	70	10	3.5	0.5		Disrupted blood-testis barrier, decreased testosterone		Decreased sperm motility, normal morphology	
	MD12032 45% fat, Mediscience Ltd	Grain-based + purified					20	35	45	1.8	2.3	Sucrose, lard, cholesterol				
(36)	824050, Special Diet Services UK	Purified	Mouse (C57BL/6)	3 wk	8 wk	4.5	20	70	10	3.5	0.5		Increased *Cyp2e1*, *Cyp19a1*, *Pparg* and *Tnf* mRNA in testis		Increased sperm DNA fragmentation	
	824053, Special Diet Services UK	Purified				3.7	20	35	45	1.8	2.3	Lard				
(37)	D12450Bi, Research Diets Inc	Purified	Mouse (C57BL/6J)	5 wk	25 wk	3.8	20	70	10	3.5	0.5	Corn starch	Increased *Pparg* mRNA, decresed *Crem*, *Dhh*, *Igf1*, *Lepr, Sh2b1* mRNA in testis		Decreased sperm motility	Decreased pregnancy rates
	D12492i, Research Diets Inc	Purified				5.2	20	20	60	1.0	3.0					
(38)	SF04-057, Specialty Feeds	Purified	Mouse (C57BL/6)	5 wk	10 wk	3.8	21	65	14	3.1	0.7		Altered testis transcriptome, decreased global methylation of testis and spermatid DNA		Altered sperm miRNAs	
	SF00-219, Specialty Feeds	Purified				4.6	17	43	40	2.5	2.4	Ghee				
(6)	SF04-057, Specialty Feeds	Purified	Mouse (C57BL/6)	6 wk	9 wk	3.8	21	65	14	3.1	0.7				Decreased sperm motility, zona binding, increased ROS, DNA fragmentation	
	SF00-219, Specialty Feeds	Purified				4.6	17	43	40	2.5	2.4	Ghee				
(39)	SF04-057, Specialty Feeds	Purified	Mouse (C57BL/6)	5 wk	18 wk	3.8	21	65	14	3.1	0.7					Decreased total embryo cell number (TE and ICM), implantations, fetal weight, crown-rump length, placental weight
	SF00-219, Specialty Feeds	Purified				4.6	17	43	40	2.5	2.4	Ghee				
(13)	SF04-057, Specialty Feeds	Purified	Mouse (C57BL/6 NHsd)	5 wk	10 wk	3.8	21	65	14	3.1	0.7				Altered sperm miRNAs	
	SF00-219, Specialty Feeds	Purified				4.6	17	43	40	2.5	2.4	Ghee				
(4)	2016 Global, Envigo	Grain-based	Mouse (C57BL/6)	10 wk	15 wk	3.0	22	66	12	3.0	0.5		Decreased *Crisp4* and *Lepr* mRNA in testis	Decreased *Crisp4* mRNA in epididymis		Decreased fertilization, pregnancy rates
	TD.88137, Envigo	Purified				4.5	15.2	42.7	42	2.8	2.8	Milk fat, sucrose				
(40)	Meat free rat and mouse chow, Specialty Feeds	Grain-based	Mouse (C57BL/6)	6 wk	10 wk	3.3	23	65	12	2.8	0.5			Increased leptin, insulin, decreased estradiol in seminal vesicle fluid, altered seminal vesicle fluid metabolite composition	Increased sperm *Cox4il* mRNA	
	SF00-219, Specialty Feeds	Purified				4.6	17	43	40	2.5	2.4	Ghee, sucrose				
(8)	2018S Global, Teklad	Grain-based	Rat (Sprague-Dawley)	“Sexually mature”	4 wk	3.1	24	58	18	2.4	0.8				Decreased sperm activities of lactate and pyruvate dehydrogenases, citrate synthase, respiratory chain complexes, decreased ATP, increased ROS	
	TD.03584, Teklad	Purified				5.4	15	27	58	1.8	3.9	Lard				
(41)	RM3, Special Diet Services	Grain-based	Mouse (C57BL/6)	11 wk	21 wk	3.3	27.3	61.2	11.5	2.2	0.4		Altered testis proteome, decreased Sertoli cell numbers, meiotic index, numbers of post-meiotic round spermatids			
	D12451, Research Diets, New Brunswick	Purified				4.7	20	35	45	1.8	2.3	Lard, sucrose				

% protein/carb/fat = % of total kcal. P:C = ratio of protein to carbohydrate (as energy) in diet. P:F = ratio of protein to fat (as energy) in diet.

Nutritional studies have shown that it is not just the amount of energy consumed that matters, but from where this energy is sourced. Most dietary energy comes from the three principal classes of macronutrients—protein, carbohydrate, and fat. Carbohydrates are the main source of metabolic fuel, protein provides amino acids for growth, repair and a minimal contribution of metabolic energy, and fats provide a concentrated source of energy. This is reflected in the energy density of each macronutrient—whereas protein and carbohydrates have around 4 kcal/g, fats contain around 9 kcal/g. This at least partially explains why a high fat diet often leads to increased adiposity, because high fat diets generally contain more calories per gram of food (**Table 1**). Consequently, it is not clear whether the effects of western diets on male reproduction result from differences in fat or differences in calories. It is thus unclear whether men trying to conceive should be advised to simply eat less or to specifically avoid fats. This distinction is important, as dietary fats are also used in androgen production (46), and therefore the message to avoid fats may actually have negative consequences for male fertility.

Although this review mainly focuses on the impact of over-nutrition on male fertility, studies of undernutrition provide a different lens to examine the overall impact of nutrition on reproduction. Two approaches are commonly used; caloric restriction and low protein diets, respectively reflecting decreased food availability and a common dietary deficiency observed in undernourished children (47). With the focus shifted from fat to protein in these models, a host of interesting findings have come to light, leading to a growing recognition of the importance of dietary protein in health and disease. In the context of male reproduction, dietary protein has been demonstrated to impact weights of reproductive organs, reproductive hormone concentrations (48), testicular architecture and occurrence of apoptosis during spermatogenesis (49), testicular expression of DNA methyltransferases, and sperm DNA methylation (50). Further, the level of protein in a father’s diet has also been shown to alter subsequent pre-implantation embryo gene expression, placental gene expression and imprinting, fetal bone growth (51), fetal and placental weights, placental structure (52), and adult offspring vascular function (53) and metabolism (50). These results highlight why it is important to consider the effects of dietary protein in addition to fats and carbohydrates when investigating the impact of dietary-induced obesity on reproductive outcomes. This is particularly important because in order to increase the percentage contribution of dietary energy of fat in the standard control versus western diet experimental design, the percentage of protein and/or carbohydrates must be decreased.

This problem of failing to consider macronutrient effects in concert is apparent from a comparison of the typical diets used in animal studies of obesity and reproduction. In addition to differences in the proportion of fat that is used to represent a “high fat” diet, the relative proportional reduction in proteins and carbohydrates also varies widely across studies (**Table 1**). Hence, while results are interpreted in the context of the change in fat content, studies are actually comparing diets which differ across their percentages of protein, carbohydrate, and fat (e.g., see **Table 1**, diet SF04-057 compared to diet SF00-219, **Figure 1**). In some cases, studies fix protein and vary only in carbohydrate and fat (7, 36, 37). In this case, control diets (fat 10% of total kcal) are high in carbohydrate (70% of total kcal) and high-fat diets (fat 45%–60% of total kcal) are low in carbohydrate (20%–35% of total kcal). This type of experimental design gives very little opportunity to disentangle the effects of different macronutrients, as it is not possible to conclude whether results are due to high fat alone, or the combination of higher fat and lower carbohydrate and/or protein. In order to better understand the impacts of different diet compositions, a new approach is required, which allows macronutrient impacts to be considered in the context of the whole diet.

Another consideration is that in an important respect, the composition of an experimental dietary treatment is not necessarily the same thing as the consumed diet, even in a no-choice paradigm. This is because an animal restricted to a nutritionally imbalanced food theoretically has the option to eat any one nutrient at the required level, albeit at the cost of over- and/or undereating other nutrients. Thus, a “low protein” experimental treatment might in reality not represent protein deficiency at all, but rather carbohydrate and/or fat surplus. Many experiments either do not measure intake, or else do not analyze the data to distinguish these possibilities.

## Moving Beyond the Western Diet; Introducing Nutritional Geometry

### Background to Nutritional Geometry and Macronutrient Balance

Nutritional Geometry (NG) is a multi-dimensional nutritional framework which assesses how macronutrient balance, rather than an individual macronutrient effect (e.g., high fat alone), impacts a given variable. Animal NG studies related to reproduction have employed a large number of diets which systematically vary across protein, carbohydrate and fat [e.g., (54, 55), **Figure 1**]. While this can make practical application logistically challenging, it offers a robust experimental design for studying the effects of macronutrient balance, which is more relevant to human obesity. Originally developed in studies of insects (56, 57), NG has since been used to study impacts of diet across a range of invertebrate taxa, particularly locusts, flies, crickets, and cockroaches. Given the adaptability of the framework, NG studies have extended to include many vertebrate species [e.g., fish (58), mice (55), companion animals (59), and non-human primates (60–62)]. Using principles which have been well established in these animal studies, Nutritional Geometry has also been directed increasingly toward human health (63, 64).

One insight to emerge from NG is the “protein leverage hypothesis”, a theory to explain why modern diets are driving the obesity epidemic (34). The PLH posits that food intake in humans is driven most strongly to fulfil a target intake for protein, which passively influences (“leverages”) the intake of non-protein energy (34, 65). A nutrient-specific appetite for protein is widespread among animal species, and evidence that this powerful protein appetite has interacted with a decline in the density of protein in the industrialized food supply to drive human obesity has accumulated rapidly in recent years [e.g., see (33)]. This is seen particularly in the modern diet of Western countries, where commonly consumed ultra-processed foods are low in protein relative to fats and carbohydrates, driving increased overall energy intake (66, 67). The impact of dietary manipulations on food intake is therefore an important consideration and should be measured in studies of nutritional effects on reproduction.

### Data Visualization Using Nutritional Geometry

One of the major advantages of the NG approach is that it provides a graphical visualization of the effects of macronutrients. Data for each response variable are mapped on to a multidimensional nutrient space, allowing for a generalized overview of how an outcome is impacted by different diet compositions (**Box 1**). This method allows the individual and interactive effects of nutrients to be explored and disentangled. Results can be interpreted on the basis of the dietary macronutrient proportions (% of total kcal from each nutrient) (68), or absolute macronutrient intakes (g or kcal eaten of each nutrient). Absolute macronutrient intakes are a function of diet composition and the amounts of food an individual consumes. The NG approach provides a platform both for examining the effects of dietary nutrient mixtures on outcomes of interest, and for developing a guide for how experimental diets can be adjusted to achieve a desired outcome. For example, using this framework, it can be seen why diets high in protein are effective for weight loss—less calories are consumed (69). However, this excess protein consumption comes at a cost—animals consuming these high protein/low carbohydrate diets show signs of metabolic disease and have shorter lifespans (70–72).

Box 1 **A guide to interpreting Nutritional Geometry surface figures.**

The figures presented in **A** and **B** represent two different experimental approaches to using Nutritional Geometry. In **(A)**, the study design incorporates a range of different diet structures (protein/carbohydrate/fat %), which vary in their energy density. Such a design will significantly impact overall energy intake and is therefore analyzed according to intake of each nutrient (typically in kJ/day). In **(B)**, the study design incorporates different diet structures, but all diets have an equal energy density (isocaloric). This design typically only has minor effects on overall energy intake and is therefore analyzed according to the amount of each nutrient in the diet (typically as percentage of total kcal in the diet).

Both figures depict the impact of macronutrient balance on a given response variable (e.g., basal glucose). A color scale is used to indicate the level of the response variable (blue = minimum, red = maximum), with isolines giving numerical values. Isoline values vary by a fixed increment and the distance between isolines indicates the magnitude of nutrient effect over a given range (distant = minimal impact, close = substantial impact). In an intake-based model like **(A)**, responses are often compared based on protein versus non-protein (carbohydrate and fat) energy. Alternatively, a 3×3 panel figure can be used to show primary interactions at the lower, median and upper quartile of the third macronutrient (e.g., protein versus carbohydrate, at the 25th, 50th and 75th percentile of fat). If appropriate to the medium, a rotating animation could be created to display all three dimensions simultaneously. In a diet-based model like **(B)**, responses are compared based on diet structure, and the impacts of all three nutrients are analyzed simultaneously. Note that for the diagonal axis (in this case fat), the value increases from 0% at the diagonal axis to 100% at the origin.

**Figure:**
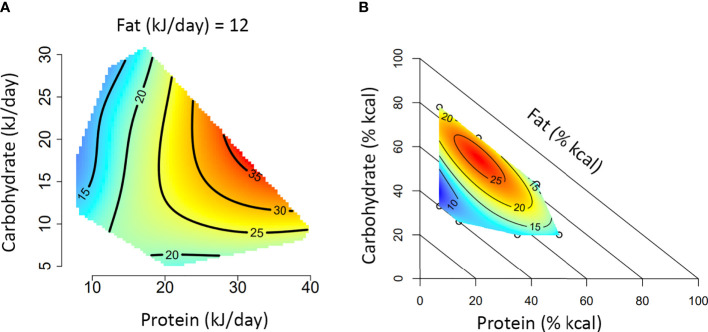

### Nutritional Geometry in Action

NG has been used to understand how diet affects different aspects of health, including trade-offs between lifespan and reproduction (55, 70, 73). In general, these studies in mice and insects show that reproductive function is optimized by diets higher in protein content than diets that maximize lifespan. However, the strength of this response differs with sex (55, 70). This may be a true effect of differing nutrient requirements for reproduction in females versus males, or it may be an artefact of difficulties in assessing reproductive function in males (or likely a combination of both). Male reproduction in insects and fish is often assessed by an indirect measure of pre-mating investment in traits such as calling effort (70, 74), pheromone expression (75, 76), size of sexually-selected traits (77), and courting behavior (58). Other studies in both insects and mice have used measures of post-mating investment including testes and accessory glands size (55), sperm number and quality (78), and mating success (75). Nutrient effects on pre- and post-mating sexual traits can differ (79), and may be age- and context-dependent (80). Therefore, to accurately measure male reproductive function, multiple measures may need to be assessed (81).

A more complete assessment of male reproductive function may also be obtained by examining offspring produced from mating trials. Insect studies examining the proportion of eggs that hatched after standardized females were mated to experimental males have found that protein has negative (82, 83) or non-linear (78) effects on male fertility. Similarly, studies in *Drosophila* assessing mating in a competitive context have found that male reproductive success is maximized on diets with intermediate levels of protein (84, 85), and a low protein to carbohydrate ratio (86). These studies suggest that while female reproduction may be enhanced by increased dietary protein, male reproduction may be enhanced on lower protein to carbohydrate ratios—a diet similar to that which maximizes lifespan and metabolic health. However, as dietary nutrients have different effects on different aspects of male reproductive function (79), dietary recommendations may need to be specific to the desired outcome. As many of these studies have shown, rarely is one macronutrient wholly responsible for an effect; instead, the balance of macronutrients has often proven to be the most significant factor determining an outcome. Therein lies the advantage of the NG approach in shifting from studying a single nutrient to the interactions of multiple nutrients.

## Important Nutritional Considerations to Improve and Expand Diet Study Designs

### Caloric Density

As discussed above, the varying caloric densities of control and western diets present an issue for the interpretation of results. When diets differ in their energy density, whether effects are derived from calories or macronutrients cannot be ascertained—a common point of contention in nutrition research (72). However, energy density can be standardized (made isocaloric) using indigestible fibre (e.g., cellulose), so that while protein, carbohydrate, and fat are at different levels, diets provide the same amount of energy per gram. Thus, isocaloric diets are a useful tool to improve the clarity of results and are commonly used in NG studies [e.g., (87)]. To study the effects of calories using the NG approach, researchers have the option of analyzing data on the basis of calories consumed (which differs only with the amount of food eaten, not the macronutrient balance). Alternatively, the study design can include a range of different macronutrient compositions at multiple calorie densities (e.g., low 3 kcal/g versus high 5 kcal/g for each combination of protein, carbohydrate, and fat).

### Macronutrient Quality

Different foods differ in their biochemical profile of amino acids, fatty acids, and carbohydrate types (88, 89). As a result, the primary dietary sources of each macronutrient are likely to be just as important as overall macronutrient balance (90, 91), and a small number of studies support that this extends to reproduction. For example, when protein is supplied at a consistent level, vervet monkeys given animal protein (milk solids) had significantly poorer semen parameters than those fed plant protein (maize and legumes) (92). Similarly, dietary fat differentially impacts testicular enzyme activity depending on whether it is derived from virgin olive oil or butter (93). While virgin olive oil (monounsaturated fat) increased dipeptidyl peptidase IV activity, helping to maintain normal spermatogenesis, butter (saturated fat) increased the activity of gamma glutamyl transpeptidase, contributing to maintenance of the intracellular glutathione pool. While research into the effects of macronutrient source on reproduction is currently limited, it is likely to play an important role in response to diet and should also be a consideration in future studies.

### Micronutrients

Beyond the macronutrients which provide dietary energy, Nutritional Geometry has also proven useful for studying the effects of dietary vitamins and minerals (79, 94). Many micronutrients, including calcium, sodium, zinc, potassium, and magnesium, have important roles in male reproduction, impacting testicular development, semen quality, and sperm biochemical processes (95). Micronutrient imbalances have also been suggested as a causal factor in unexplained female infertility (96). Reduced intake of dietary antioxidants including lycopene, vitamin C, folate, and carotenoids, has been associated with poorer semen parameters (97, 98). In addition, iodine intake outside the recommended range in men has been associated with increased time to conception (99), supporting a critical role for micronutrient balance in fertility. Supplementation of micronutrients, particularly those with antioxidant activity (e.g., vitamins C and E, selenium), has been widely studied as a tool for improving reproductive outcomes of infertile men (100, 101). However, results have varied widely depending on the andrological diagnosis and the type, quantity and duration of micronutrient supplementation. While micronutrients clearly play important roles in male reproductive physiology, there have been no studies which systematically evaluate the impacts of dietary micronutrient intake on reproductive success. Micronutrients are particularly important to consider in the context of obesity, as micronutrient deficiency appears to be common in obese individuals (102, 103). Further, micronutrient supplementation may be able to limit negative effects of obesity on sperm function (104). Overall, there is an ongoing need for systematic research into how dietary micronutrients impact reproduction in both lean and obese males.

### Dietary Restriction

In studies using both western diets and a NG approach, food is generally provided *ad libitum*. However, another approach used in dietary studies is to restrict either the amount or timing of access to food. There is good evidence that the temporal pattern of intake, including caloric restriction, periodic and intermittent fasting can have important effects in addition to those of diet composition (105). In relation to male reproduction, caloric restriction has been demonstrated to impact testicular gene expression (106, 107), including expression of leptin and ghrelin receptors (108). Intermittent fasting has also been shown to affect testicular gene expression (106), as well as testosterone production (106, 109, 110). As caloric restriction and intermittent fasting remain popular (111) and recommended (112) weight loss strategies, future studies should also seek to investigate the effects of different intake patterns on reproductive health.

### Genetics and the Human Context

One important consideration which spans both nutrition and reproductive biology, is the contribution of genetics. There are genetic factors, including copy number variants, gene mutations, single nucleotide polymorphisms and chromosomal abnormalities, implicated in obesity (113), and male infertility (114, 115). In the context of obesity, there is also the important consideration of nutrigenomics; the influence of nutrients themselves on gene expression (116). Given the important contribution of a unique genetic background to both response to diet and male fertility, this is a factor which poses a significant limitation in current studies. While C57BL/6 mice are used extensively as a model species in obesity research, this is an inbred sub-strain with limited genetic variability. Further, the consistent diet offered in animal studies does not reflect the depth of dietary variation in humans. While both constraints are inherent limitations of animal studies, they highlight the importance of moving from a single model to a variety of models (e.g., different mouse strains, non-human primates), and eventually to human studies. Making such a transition can allow for analyses based on populations with higher genetic variability. Further, Nutritional Geometry can be used to extend findings of animal studies into more complex human dietary patterns, analyzing either free-choice feeding from a selected range of foods (65) or dietary survey data (117).

## Features of Study Design and Measurement Specific to Male Reproduction

Investigations into how diet and obesity impact male reproductive function require the collaboration of two distinct research fields; nutrition and reproductive biology. In addition to considering the dietary aspect of animal studies, issues specific to studying male reproduction need to be considered. The first of these is the timing and length of diet treatments, which vary considerably in previous western diet-based studies (**Table 1**). In agricultural species, a significant body of research shows that many effects of diet (including over and under feeding) observed in pre-pubertal males are different in sexually mature males (118, 119). In terms of treatment length, many studies apply dietary interventions for a minimum of one complete spermatogenic cycle [34.5 days in mice (120), 56 days in rats (121)], to ensure that mature spermatozoa in the ejaculate are “exposed” to treatment throughout the entirety of spermatogenesis. Conversely, some recent studies have indicated impacts of diet on sperm function in the short term (<2 weeks) (104, 122). There is no wrong answer here in terms of when to start and stop treatment, but the interpretation of results should consider whether treatments were applied pre or post-puberty, and how treatment duration relates to sperm development.

Another important consideration is the measurement of reproductive function. Fertility in humans is simply defined as natural conception within 12 months of unprotected intercourse (123), with time to conception commonly used to describe an individual’s likelihood of fertility (11). Previous diet studies have employed a range of assessments, including basic observational measurements [e.g., testis size (55), sperm motility, histology (7)], molecular biology assays [e.g., miRNAseq (12), proteomics (41), enzyme activity (8), oxidative stress markers (6)], and direct measures of conception success [i.e., fertilization rate, blastulation rate, pregnancy rate (37, 39)]. While no one assay provides an infallible measurement of fertility, the combination of several variables will help to build a clearer picture of how diet and obesity impact male reproduction overall.

Finally, there should be a concerted effort to both capture and understand the unique impacts of diet on male compared to female reproductive physiology. It may be tempting to conclude that overall effects of diet on reproduction (i.e., increases or decreases in fertility) observed in one sex are equally applicable to the other sex. However, as discussed above, female and male reproductive performance appear to be optimized on different diets. Further, studies have indicated that female and male reproductive traits are differentially impacted by the same macronutrient ratios (55, 82, 83, 124). For example, male mice consuming a diet with an equal ratio of protein to carbohydrate had the largest testes and seminal vesicles, whereas female mice consuming the same diet had the largest uteri, but frequency of estrus, total follicle count and number of corpora lutea were reduced (55). Ultimately, future studies should endeavour to compare and contrast female and male reproductive responses to diet in order to determine whether the ideal macronutrient ratio to support reproduction is sex-specific.

## Discussion

As the prevalence of obesity continues to rise, and more negative implications for male reproductive physiology are discovered, its continued study remains a high priority. So far, research has provided strong evidence that a high fat diet negatively impacts male reproduction. However, as posited by the protein leverage hypothesis (34), dietary macronutrient balance rather than fat alone is likely to account for rising levels of obesity in the human population. In this context, the approach that is used to study obesity and the extent to which it captures the reality of the human experience must be considered. Using a tool such as Nutritional Geometry to study many different macronutrient combinations will not only provide information on which diets are detrimental but could also help guide research toward diets which may support reproductive function. This concept is particularly relevant when considering our approach to providing nutritional advice to men who are interested in conceiving.

Despite the observed impacts of obesity on male reproduction and the fact that men report >80% of pregnancies are planned (125), widespread, professional pre-conception nutritional guidance for men remains almost non-existent (126, 127). The advice most commonly given and acted upon by men is to lose weight and eat a healthy diet (126). This is undoubtedly good advice, given the clear negative impacts of obesity on male reproduction (18, 20) and the strong relationship between diet and obesity risk (34, 128). However, there is no clear definition of what a “healthy diet” for reproduction is.

Switching to a “healthy diet” for most men means reducing intakes of foods containing saturated fat and added salt and sugars, and eating a wider variety of unprocessed foods (as recommended by nutritional dietary guidelines). While nutritional guidelines from different countries also give recommendations for macronutrient proportions (e.g., USA; 10%–35% protein, 45%–65% carbohydrate, 20%–35% fat as % of total kcal) (129), it remains unclear whether this diet structure is optimal for male fertility. While observational studies in humans have identified associations between dietary patterns and semen quality (130), the ideal macronutrient balance to support male reproduction is far from being well defined. Importantly, this is not necessarily the same as a diet which supports overall health and longevity, nor the same diet which supports female reproduction (55). In addition, it is not clear whether different advice is required in different contexts—taking other extrinsic, intrinsic, and genetic factors into account. There is a clear need to further explore how diet impacts male reproductive function in order to develop evidence-based pre-conception nutritional guidance for men.

There are many exciting potential applications of Nutritional Geometry in the landscape of male reproduction, covering both fundamental and applied aspects of reproductive research. Beginning with fundamental research conducted in rodent models, results would inform more targeted pre-clinical animal research, as well as nutritional intervention based clinical trials in humans. The information gathered by this approach would provide strong evidence on which to build pre-conception guidelines. On a fundamental level, NG can be used to explore which macronutrient ratio best supports male reproduction, and whether this differs from a) what supports female reproduction and b) what supports overall health. As the impacts of a paternal high fat diet on offspring health and reproduction are rapidly being uncovered (131), NG will likely be useful in exploring new avenues of paternal effects. NG may also be useful in exploring the effects of macronutrient source and weight loss strategies (e.g., caloric restriction, intermittent fasting) on reproductive function, and whether these factors alter the ideal macronutrient ratio. In the human context, it will be important to determine the impact of differing treatment durations to establish whether diet changes within the relatively short term pre-conception planning window (<12 months) are a feasible strategy. With the rising use of assisted reproductive technologies (e.g., IVF) for conception (132), more clinically focused research could use NG to examine whether the ideal dietary macronutrient ratio to support reproduction is applicable outside of natural conception.

Animal studies have used high fat and western diets for decades in the pursuit of understanding the many consequences of obesity. While this approach has produced a wealth of information on the physiological impacts of obesity, it doesn’t tell the whole story and limits what interpretations can be made about the role of diet. Nutritional Geometry shifts the focus from the effect of fat alone to complex and interacting effects of dietary macronutrient balance. Adopting the NG approach in future studies will provide more information on how the overall diet composition impacts male reproduction. In turn, this will allow for the development of evidence-based pre-conception nutritional guidelines for men, to support natural conception and potentially limit negative effects on offspring.

## Author Contributions

TP and AC conceived the review. TP, DR, SS, and AC wrote and reviewed the paper. All authors contributed to the article and approved the submitted version.

## Funding

Authors are supported by research grants from Novo Nordisk (Foundation Grant NNF18OC0033754) and the NHMRC (Program Grant GNT1149976). Article Processing Charges were paid by the University of Sydney through an equity award to AC.

## Conflict of Interest

The authors declare that the research was conducted in the absence of any commercial or financial relationships that could be construed as a potential conflict of interest.

## References

[1] Hales C Carroll M Fryar C Ogden C. Prevalence of obesity among adults and youth: United States, 2015–2016. Natl Center Health Stat Data Brief (2017) 288:1–8. 29155689

[2] Australian Institute of Health and Welfare. Overweight and obesity: An interactive insight. Canberra: AIHW (2019).

[3] National Statistics. Health Survey for England 2017: Adult and Child Overweight and Obesity. England: NHS UK (2018).

[4] Borges BC Garcia-Galiano D da Silveira Cruz-Machado S Han X Gavrilina GB Saunders TL. Obesity-induced infertility in male mice is associated with disruption of *Crisp4* expression and sperm fertilization capacity. Endocrinology (2017) 158:2930–43. 10.1210/en.2017-00295 PMC5659670 28911169

[5] Pini T Parks J Russ J Dzieciatkowska M Hansen KC Schoolcraft WB. Obesity significantly alters the human sperm proteome, with potential implications for fertility. J Assisted Reprod Genet (2020) 37:777–87. 10.1007/s10815-020-01707-8 PMC7183029 32026202

[6] Bakos HW Mitchell M Setchell BP Lane M. The effect of paternal diet-induced obesity on sperm function and fertilization in a mouse model. Int J Androl (2011) 34:402–10. 10.1111/j.1365-2605.2010.01092.x 20649934

[7] Fan Y Liu Y Xue K Gu G Fan W Xu Y. Diet-induced obesity in male C57BL/6 mice decreases fertility as a consequence of disrupted blood-testis barrier. PLoS One (2015) 10:e0120775. 10.1371/journal.pone.0120775 25886196 PMC4401673

[8] Ferramosca A Conte A Moscatelli N Zara V. A high-fat diet negatively affects rat sperm mitochondrial respiration. Andrology (2016) 4:520–5. 10.1111/andr.12182 27062222

[9] Salas-Huetos A Maghsoumi-Norouzabad L James ER Carrell DT Aston KI Jenkins TG. Male adiposity, sperm parameters and reproductive hormones: An updated systematic review and collaborative meta-analysis. Obes Rev (2021) 22:e13082. 10.1111/obr.13082 32705766

[10] Ramlau-Hansen CH Thulstrup AM Nohr EA Bonde JP Sørensen TIA Olsen J. Subfecundity in overweight and obese couples. Hum Reprod (2007) 22:1634–7. 10.1093/humrep/dem035 17344224

[11] Sallmén M Sandler DP Hoppin JA Blair A Baird DD. Reduced fertility among overweight and obese men. Epidemiology (2006) 17:520–3. 10.1097/01.ede.0000229953.76862.e5 16837825

[12] de Castro Barbosa T Ingerslev LR Alm PS Versteyhe S Massart J Rasmussen M. High-fat diet reprograms the epigenome of rat spermatozoa and transgenerationally affects metabolism of the offspring. Mol Metab (2016) 5:184–97. 10.1016/j.molmet.2015.12.002 PMC4770269 26977389

[13] Fullston T Ohlsson-Teague EMC Print CG Sandeman LY Lane M. Sperm microRNA content is altered in a mouse model of male obesity, but the same suite of microRNAs are not altered in offspring’s sperm. PLoS One (2016) 11:e0166076. 10.1371/journal.pone.0166076 27814400 PMC5096664

[14] Reijo R Lee T-Y Salo P Alagappan R Brown LG Rosenberg M. Diverse spermatogenic defects in humans caused by Y chromosome deletions encompassing a novel RNA–binding protein gene. Nat Genet (1995) 10:383–93. 10.1038/ng0895-383 7670487

[15] Rochebrochard E Thonneau P. Paternal age ≥40 years: An important risk factor for infertility. Am J Obstet Gynecol (2003) 189:901–5. 10.1067/S0002-9378(03)00753-1 14586322

[16] Lin CM Chang WP Doyle P Wang JD Lee LT Lee CL. Prolonged time to pregnancy in residents exposed to ionising radiation in cobalt-60-contaminated buildings. Occup Environ Med (2010) 67:187–95. 10.1136/oem.2008.045260 19773284

[17] Harlev A Agarwal A Gunes SO Shetty A du Plessis,SS. Smoking and male infertility: An evidence-based review. World J Men’s Health (2015) 33:143–60. 10.5534/wjmh.2015.33.3.143 PMC4709430 26770934

[18] Chambers TJG Richard RA. The impact of obesity on male fertility. Hormones (2015) 14:563–8. 10.14310/horm.2002.1621 26732149

[19] Craig JR Jenkins TG Carrell DT Hotaling JM. Obesity, male infertility, and the sperm epigenome. Fertil Steril (2017) 107:848–59. 10.1016/j.fertnstert.2017.02.115 28366411

[20] Raad G Hazzouri M Bottini S Trabucchi M Azoury J Grandjean V. Paternal obesity: How bad is it for sperm quality and progeny health? Basic Clin Androl (2017) 27:20. 10.1186/s12610-017-0064-9 29123667 PMC5657098

[21] Chavarro JE Toth TL Wright DL Meeker JD Hauser R. Body mass index in relation to semen quality, sperm DNA integrity, and serum reproductive hormone levels among men attending an infertility clinic. Fertil Steril (2010) 93:2222–31. 10.1016/j.fertnstert.2009.01.100 PMC2864498 19261274

[22] Eisenberg ML Kim S Chen Z Sundaram R Schisterman EF Buck Louis GM. The relationship between male BMI and waist circumference on semen quality: data from the LIFE study. Hum Reprod (2014) 29:193–200. 10.1093/humrep/det428 24306102 PMC3896223

[23] McPherson NO Tremellen K. Increased BMI ‘alone’ does not negatively influence sperm function - a retrospective analysis of men attending fertility treatment with corresponding liver function results. Obes Res Clin Pract (2020) 14:164–7. 10.1016/j.orcp.2020.03.003 32321679

[24] Reis LO Dias FGF. Male fertility, obesity, and bariatric surgery. Reprod Sci (2012) 19:778–85. 10.1177/1933719112440053 22534334

[25] Thomsen L Humaidan P Bungum L Bungum M. The impact of male overweight on semen quality and outcome of assisted reproduction. Asian J Androl (2014) 16:749–54. 10.4103/1008-682X.125398 PMC4215681 24759576

[26] Hruby A Manson JE Qi L Malik VS Rimm EB Sun Q. Determinants and consequences of obesity. Am J Public Health (2016) 106:1656–62. 10.2105/AJPH.2016.303326 PMC4981805 27459460

[27] Bray GA Paeratakul S Popkin BM. Dietary fat and obesity: A review of animal, clinical and epidemiological studies. Physiol Behav (2004) 83:549–55. 10.1016/j.physbeh.2004.08.039 15621059

[28] Bray GA Popkin BM. Dietary fat intake does affect obesity! Am J Clin Nutr (1998) 68:1157–73. 10.1093/ajcn/68.6.1157 9846842

[29] Field AE Willett WC Lissner L Colditz GA. Dietary fat and weight gain among women in the Nurses’ Health Study. Obesity (2007) 15:967–76. 10.1038/oby.2007.616 17426332

[30] Willett W. Dietary fat and obesity: An unconvincing relation. Am J Clin Nutr (1998) 68:1149–50. 10.1093/ajcn/68.6.1149 9846838

[31] Willett WC. Dietary fat and obesity: Lack of an important role. Scand J Nutr (2003) 47:58–67. 10.1080/11026480310007953

[32] Hall KD. The Potential Role of Protein Leverage in the US Obesity Epidemic. Obesity (2019) 27:1222–4. 10.1002/oby.22520 PMC7147114 31095898

[33] Raubenheimer D Simpson SJ. Protein leverage: Theoretical foundations and ten points of clarification. Obesity (2019) 27:1225–38. 10.1002/oby.22531 31339001

[34] Simpson SJ Raubenheimer D. Obesity: The protein leverage hypothesis. Obes Rev (2005) 6:133–42. 10.1111/j.1467-789X.2005.00178.x 15836464

[35] Pellizzon MA Ricci MR. Choice of laboratory rodent diet may confound data interpretation and reproducibility. Curr Dev Nutr (2020) 4:nzaa031. 10.1093/cdn/nzaa031 32258990 PMC7103427

[36] Duale N Steffensen IL Andersen J Brevik A Brunborg G Lindeman B. Impaired sperm chromatin integrity in obese mice. Andrology (2014) 2:234–43. 10.1111/j.2047-2927.2013.00178.x 24459046

[37] Ghanayem BI Bai R Kissling GE Travlos G Hoffler U. Diet-induced obesity in male mice is associated with reduced fertility and potentiation of acrylamide-induced reproductive toxicity. Biol Reprod (2009) 82:96–104. 10.1095/biolreprod.109.078915 19696015 PMC2802115

[38] Fullston T Ohlsson Teague EMC Palmer NO DeBlasio MJ Mitchell M Corbett M. Paternal obesity initiates metabolic disturbances in two generations of mice with incomplete penetrance to the F2 generation and alters the transcriptional profile of testis and sperm microRNA content. FASEB J (2013) 27:4226–43. 10.1096/fj.12-224048 23845863

[39] McPherson NO Bakos HW Owens JA Setchell BP Lane M. Improving metabolic health in obese male mice via diet and exercise restores embryo development and fetal growth. PLoS One (2013) 8:e71459. 10.1371/journal.pone.0071459 23977045 PMC3747240

[40] Binder NK Sheedy JR Hannan NJ Gardner DK. Male obesity is associated with changed spermatozoa Cox4i1 mRNA level and altered seminal vesicle fluid composition in a mouse model. Mol Hum Reprod (2015) 21:424–34. 10.1093/molehr/gav010 25731709

[41] Jarvis S Gethings LA Samanta L Pedroni SMA Withers DJ Gray N. High fat diet causes distinct aberrations in the testicular proteome. Int J Obes (2020) 44:1958–69. 10.1038/s41366-020-0595-6 PMC7445115 32678325

[42] McPherson NO Owens JA Fullston T Lane M. Preconception diet or exercise intervention in obese fathers normalizes sperm microRNA profile and metabolic syndrome in female offspring. Am J Physiol Endocrinol Metabol (2015) 308:E805–21. 10.1152/ajpendo.00013.2015 25690453

[43] Fullston T Palmer NO Owens JA Mitchell M Bakos HW Lane M. Diet-induced paternal obesity in the absence of diabetes diminishes the reproductive health of two subsequent generations of mice. Hum Reprod (2012) 27:1391–400. 10.1093/humrep/des030 22357767

[44] Mitchell M Bakos HW Lane M. Paternal diet-induced obesity impairs embryo development and implantation in the mouse. Fertil Steril (2011) 95:1349–53. 10.1016/j.fertnstert.2010.09.038 21047633

[45] Palmer NO Fullston T Mitchell M Setchell BP Lane M. SIRT6 in mouse spermatogenesis is modulated by diet-induced obesity. Reprod Fertil Dev (2011) 23:929–39. 10.1071/RD10326 21871212

[46] Gromadzka-Ostrowska J. Effects of dietary fat on androgen secretion and metabolism. Reprod Biol (2006) 6:13–20. 17220937

[47] Salameh E Morel FB Zeilani M Déchelotte P Marion-Letellier R. Animal models of undernutrition and enteropathy as tools for assessment of nutritional intervention. Nutrients (2019) 11:2233. 10.3390/nu11092233 PMC6770013 31527523

[48] Ajuogu PK Al-Aqbi MA Hart RA Wolden M Smart NA McFarlane JR. The effect of dietary protein intake on factors associated with male infertility: A systematic literature review and meta-analysis of animal clinical trials in rats. Nutr Health (2020) 26:53–64. 10.1177/0260106019900731 31992124

[49] Morgan HL Ampong I Eid N Rouillon C Griffiths HR Watkins AJ. Low protein diet and methyl-donor supplements modify testicular physiology in mice. Reproduction (2020) 159:627–41. 10.1530/REP-19-0435 PMC7159163 32163913

[50] Watkins AJ Dias I Tsuro H Allen D Emes RD Moreton J. Paternal diet programs offspring health through sperm- and seminal plasma-specific pathways in mice. Proc Natl Acad Sci U S A (2018) 115:10064. 10.1073/pnas.1806333115 30150380 PMC6176621

[51] Watkins AJ Sirovica S Stokes B Isaacs M Addison O Martin RA. Paternal low protein diet programs preimplantation embryo gene expression, fetal growth and skeletal development in mice. Biochim Biophys Acta (2017) 1863:1371–81. 10.1016/j.bbadis.2017.02.009 28189722

[52] Morgan HL Aljumah A Rouillon C Watkins AJ. Paternal low protein diet and the supplementation of methyl-donors impact fetal growth and placental development in mice. Placenta (2021) 103:124–33. 10.1016/j.placenta.2020.10.020 PMC7907633 33120048

[53] Morgan HL Paganopoulou P Akhtar S Urquhart N Philomin R Dickinson Y. Paternal diet impairs F1 and F2 offspring vascular function through sperm and seminal plasma specific mechanisms in mice. J Physiol (2020) 598:699–715. 10.1113/JP278270 31617219

[54] Paris V Solon-Biet S Senior A Edwards M Desai R Tedla N. Defining the impact of dietary macronutrient balance on PCOS traits. Nat Commun (2020) 11:5262. 10.1038/s41467-020-19003-5 33067453 PMC7568581

[55] Solon-Biet SM Walters KA Simanainen UK McMahon AC Ruohonen K Ballard JWO. Macronutrient balance, reproductive function, and lifespan in aging mice. Proc Natl Acad Sci U S A (2015) 112:3481–6. 10.1073/pnas.1422041112 PMC4371964 25733862

[56] Raubenheimer D Simpson SJ. The geometry of compensatory feeding in the locust. Anim Behav (1993) 45:953–64. 10.1006/anbe.1993.1114

[57] Simpson SJ Raubenheimer D. A multi-level analysis of feeding behaviour: The geometry of nutritional decisions. Philos Trans R Soc Lond Ser B: Biol Sci (1993) 342:381–402. 10.1098/rstb.1993.0166

[58] Moatt J Fyfe M Heap E Mitchell L Moon F Walling C. Reconciling nutritional geometry with classical dietary restriction: Effects of nutrient intake, not calories, on survival and reproduction. Aging Cell (2019) 18:e12868. 10.1111/acel.12868 30456818 PMC6352320

[59] Hewson-Hughes AK Hewson-Hughes VL Colyer A Miller AT McGrane SJ Hall SR. Geometric analysis of macronutrient selection in breeds of the domestic dog, *Canis lupus familiaris*. Behav Ecol (2013) 24:293–304. 10.1093/beheco/ars168 23243377 PMC3518205

[60] Cui Z-W Wang Z-L Shao Q Raubenheimer D Lu J-Q. Macronutrient signature of dietary generalism in an ecologically diverse primate in the wild. Behav Ecol (2018) 29:804–13. 10.1093/beheco/ary003

[61] Guo S-T Hou R Garber PA Raubenheimer D Righini N Ji W-H. Nutrient-specific compensation for seasonal cold stress in a free-ranging temperate colobine monkey. Funct Ecol (2018) 32:2170–80. 10.1111/1365-2435.13134

[62] Rothman JM Raubenheimer D Chapman CA. Nutritional geometry: gorillas prioritize non-protein energy while consuming surplus protein. Biol Lett (2011) 7:847–9. 10.1098/rsbl.2011.0321 PMC3210651 21632622

[63] Raubenheimer D Simpson SJ. Nutritional Ecology and Human Health. Annu Rev Nutr (2016) 36:603–26. 10.1146/annurev-nutr-071715-051118 27296501

[64] Simpson SJ Le Couteur DG James DE George J Gunton JE Solon-Biet SM. The Geometric Framework for Nutrition as a tool in precision medicine. Nutr Healthy Aging (2017) 4:217–26. 10.3233/NHA-170027 PMC5734128 29276791

[65] Simpson SJ Batley R Raubenheimer D. Geometric analysis of macronutrient intake in humans: The power of protein? Appetite (2003) 41:123–40. 10.1016/S0195-6663(03)00049-7 14550310

[66] Hall KD Ayuketah A Brychta R Cai H Cassimatis T Chen KY. Ultra-processed diets cause excess calorie intake and weight gain: An inpatient randomized controlled trial of ad libitum food intake. Cell Metab (2019) 30:67–77.e3. 10.1016/j.cmet.2019.05.008 31105044 PMC7946062

[67] Martínez Steele E Raubenheimer D Simpson SJ Baraldi LG Monteiro CA. Ultra-processed foods, protein leverage and energy intake in the USA. Public Health Nutr (2018) 21:114–24. 10.1017/S1368980017001574 PMC10260799 29032787

[68] Raubenheimer D. Toward a quantitative nutritional ecology: The right-angled mixture triangle. Ecol Monogr (2011) 81:407–27. 10.1890/10-1707.1

[69] Freedman MR King J Kennedy E. Popular diets: A scientific review. Obes Res (2001) 9:1s–40s. 10.1038/oby.2001.113 11374180

[70] Maklakov AA Simpson SJ Zajitschek F Hall MD Dessmann J Clissold F. Sex-specific fitness effects of nutrient intake on reproduction and lifespan. Curr Biol (2008) 18:1062–6. 10.1016/j.cub.2008.06.059 18635354

[71] Mittendorfer B Klein S Fontana L. A word of caution against excessive protein intake. Nat Rev Endocrinol (2020) 16:59–66. 10.1038/s41574-019-0274-7 31728051

[72] Simpson SJ Le Couteur DG Raubenheimer D Solon-Biet SM Cooney GJ Cogger VC. Dietary protein, aging and nutritional geometry. Ageing Res Rev (2017) 39:78–86. 10.1016/j.arr.2017.03.001 28274839

[73] Lee KP Simpson SJ Clissold FJ Brooks R Ballard JWO Taylor PW. Lifespan and reproduction in *Drosophila*: New insights from nutritional geometry. Proc Natl Acad Sci U S A (2008) 105:2498. 10.1073/pnas.0710787105 18268352 PMC2268165

[74] Rapkin J Archer CR Grant CE Jensen K House CM Wilson AJ. Little evidence for intralocus sexual conflict over the optimal intake of nutrients for life span and reproduction in the black field cricket *Teleogryllus commodus*. Evolution (2017) 71:2159–77. 10.1111/evo.13299 PMC5599978 28640400

[75] Rapkin J Jensen K House CM Sakaluk SK Sakaluk JK Hunt J. The complex interplay between macronutrient intake, cuticular hydrocarbon expression and mating success in male decorated crickets. J Evol Biol (2017) 30:711–27. 10.1111/jeb.13036 28029711

[76] South SH House CM Moore AJ Simpson SJ Hunt J. Male cockroaches prefer a high carbohydrate diet that makes them more attractive to females: Implications for the study of condition dependence. Evolution (2011) 65:1594–606. 10.1111/j.1558-5646.2011.01233.x 21644951

[77] Sentinella AT Crean AJ Bonduriansky R. Dietary protein mediates a trade-off between larval survival and the development of male secondary sexual traits. Funct Ecol (2013) 27:1134–44. 10.1111/1365-2435.12104

[78] Bunning H Rapkin J Belcher L Archer CR Jensen K Hunt J. Protein and carbohydrate intake influence sperm number and fertility in male cockroaches, but not sperm viability. Proc R Soc Lond Ser B: Biol Sci (2015) 282:20142144. 10.1098/rspb.2014.2144 PMC4344140 25608881

[79] Ng SH Simpson SJ Simmons LW. Macronutrients and micronutrients drive trade-offs between male pre- and postmating sexual traits. Funct Ecol (2018) 32:2380–94. 10.1111/1365-2435.13190

[80] Macartney EL Crean AJ Bonduriansky R. Adult dietary protein has age- and context-dependent effects on male post-copulatory performance. J Evol Biol (2017) 30:1633–43. 10.1111/jeb.13087 28386961

[81] Moatt JP Nakagawa S Lagisz M Walling CA. The effect of dietary restriction on reproduction: a meta-analytic perspective. BMC Evol Biol (2016) 16:199. 10.1186/s12862-016-0768-z 27717308 PMC5054627

[82] Bonduriansky R Runagall-McNaull A Crean AJ. The nutritional geometry of parental effects: Maternal and paternal macronutrient consumption and offspring phenotype in a neriid fly. Funct Ecol (2016) 30:1675–86. 10.1111/1365-2435.12643

[83] Zajitschek F Zajitschek SRK Friberg U Maklakov AA. Interactive effects of sex, social environment, dietary restriction, and methionine on survival and reproduction in fruit flies. AGE (2013) 35:1193–204. 10.1007/s11357-012-9445-3 PMC3705097 22798158

[84] Fricke C Bretman A Chapman T. Adult male nutrition and reproductive success in *Drosophila melanogaster*. Evolution (2008) 62:3170–7. 10.1111/j.1558-5646.2008.00515.x 18786187

[85] Reddiex AJ Gosden TP Bonduriansky R Chenoweth SF. Sex-specific fitness consequences of nutrient intake and the evolvability of diet preferences. Am Nat (2013) 182:91–102. 10.1086/670649 23778229

[86] Jensen K McClure C Priest NK Hunt J. Sex-specific effects of protein and carbohydrate intake on reproduction but not lifespan in *Drosophila melanogaster*. Aging Cell (2015) 14:605–15. 10.1111/acel.12333 PMC4531074 25808180

[87] Solon-Biet S McMahon A Ballard J Ruohonen K Wu L Cogger V. The Ratio of Macronutrients, Not Caloric Intake, Dictates Cardiometabolic Health, Aging, and Longevity in Ad Libitum-Fed Mice. Cell Metab (2014) 19:418–30. 10.1016/j.cmet.2014.02.009 PMC5087279 24606899

[88] Hoffman JR Falvo MJ. Protein - Which is best? J Sports Sci Med (2004) 3:118–30. PMC3905294 24482589

[89] Orsavova J Misurcova L Ambrozova JV Vicha R Mlcek J. Fatty acids composition of vegetable oils and its contribution to dietary energy intake and dependence of cardiovascular mortality on dietary intake of fatty acids. Int J Mol Sci (2015) 16:12871–90. 10.3390/ijms160612871 PMC4490476 26057750

[90] Solon-Biet SM Cogger VC Pulpitel T Wahl D Clark X Bagley E. Branched chain amino acids impact health and lifespan indirectly via amino acid balance and appetite control. Nat Metab (2019) 1:532–45. 10.1038/s42255-019-0059-2 PMC6814438 31656947

[91] Wali JA Raubenheimer D Senior AM Le Couteur DG Simpson SJ. Cardio-metabolic consequences of dietary carbohydrates: reconciling contradictions using nutritional geometry. Cardiovasc Res (2021) 117:386–401. 10.1093/cvr/cvaa136 32386289

[92] Johnson Q Veith W. Effect of dietary plant and animal protein intake on sperm quality in monkeys. Arch Androl (2001) 46:145–51. 10.1080/01485010151094092 11297069

[93] Domínguez-Vías G Segarra A Martínez-Cañamero M Ramírez-Sanchez M Prieto I. Influence of a virgin olive oil versus butter plus cholesterol-enriched diet on testicular enzymatic activities in adult male rats. Int J Mol Sci (2017) 18:1701. 10.3390/ijms18081701 PMC5578091 28777292

[94] Harrison SJ Raubenheimer D Simpson SJ Godin JG Bertram SM. Towards a synthesis of frameworks in nutritional ecology: Interacting effects of protein, carbohydrate and phosphorus on field cricket fitness. Proc Biol Sci (2014) 281:20140539. 10.1098/rspb.2014.0539 25143029 PMC4150310

[95] Skoracka K Eder P Łykowska-Szuber L Dobrowolska A Krela-Kaźmierczak I. Diet and nutritional factors in male (in)fertility-underestimated factors. J Clin Med (2020) 9:1400. 10.3390/jcm9051400 PMC7291266 32397485

[96] Noventa M Quaranta M Vitagliano A Cinthya V Valentini R Campagnaro T. May underdiagnosed nutrition imbalances be responsible for a portion of so-called unexplained infertility?From diagnosis to potential treatment options. Reprod Sci (2016) 23:812–22. 10.1177/1933719115620496 26692540

[97] Mendiola J Torres-Cantero AM Vioque J Moreno-Grau JM Ten J Roca M. A low intake of antioxidant nutrients is associated with poor semen quality in patients attending fertility clinics. Fertil Steril (2010) 93:1128–33. 10.1016/j.fertnstert.2008.10.075 19147135

[98] Zareba P Colaci DS Afeiche M Gaskins AJ Jørgensen N Mendiola J. Semen quality in relation to antioxidant intake in a healthy male population. Fertil Steril (2013) 100:1572–9. 10.1016/j.fertnstert.2013.08.032 PMC3843991 24094424

[99] Sun Y Chen C Liu GG Wang M Shi C Yu G. The association between iodine intake and semen quality among fertile men in China. BMC Public Health (2020) 20:461. 10.1186/s12889-020-08547-2 32252717 PMC7137216

[100] Buhling JK Laakmann JE. The effect of micronutrient supplements on male fertility. Curr Opin Obstet Gynecol (2014) 26:199–209. 10.1097/GCO.0000000000000063 24759120

[101] Buhling K Schumacher A Eulenburg CZ Laakmann E. Influence of oral vitamin and mineral supplementation on male infertility: A meta-analysis and systematic review. Reprod Biomed Online (2019) 39:269–79. 10.1016/j.rbmo.2019.03.099 31160241

[102] Krzizek E-C Brix J Herz C Kopp H Schernthaner G-H Schernthaner G. Prevalence of micronutrient deficiency in patients with morbid obesity before bariatric surgery. Obes Surg (2018) 28:643–8. 10.1007/s11695-017-2902-4 28849358

[103] McKay J Ho S Jane M Pal S. Overweight & obese Australian adults and micronutrient deficiency. BMC Nutr (2020) 6:12–2. 10.1186/s40795-020-00336-9 PMC7193396 32377370

[104] McPherson N Shehadeh H Fullston T Zander-Fox D Lane M. Dietary micronutrient supplementation for 12 days in obese male mice restores sperm oxidative stress. Nutrients (2019) 11:2196. 10.3390/nu11092196 PMC6770166 31547309

[105] Di Francesco A Di Germanio C Bernier M de Cabo R. A time to fast. Science (2018) 362:770–5. 10.1126/science.aau2095 PMC8504313 30442801

[106] Martin B Pearson M Brenneman R Golden E Wood W Prabhu V. Gonadal transcriptome alterations in response to dietary energy intake: Sensing the reproductive environment. PLoS One (2009) 4:e4146. 10.1371/journal.pone.0004146 19127293 PMC2607546

[107] Sharov AA Falco G Piao Y Poosala S Becker KG Zonderman AB. Effects of aging and calorie restriction on the global gene expression profiles of mouse testis and ovary. BMC Biol (2008) 6:24. 10.1186/1741-7007-6-24 18522719 PMC2426674

[108] Martins AD Jarak I Morais T Carvalho RA Oliveira PF Monteiro MP. Caloric restriction alters the hormonal profile and testicular metabolome, resulting in alterations of sperm head morphology. Am J Physiol Endocrinol Metabol (2020) 318:E33–43. 10.1152/ajpendo.00355.2019 31770015

[109] Kumar S Kaur G. Intermittent fasting dietary restriction regimen negatively influences reproduction in young rats: A study of hypothalamo-hypophysial-gonadal axis. PLoS One (2013) 8:e52416. 10.1371/journal.pone.0052416 23382817 PMC3558496

[110] Lynn SE Stamplis TB Barrington WT Weida N Hudak CA. Food, stress, and reproduction: Short-term fasting alters endocrine physiology and reproductive behavior in the zebra finch. Horm Behav (2010) 58:214–22. 10.1016/j.yhbeh.2010.03.015 20362578

[111] Obert J Pearlman M Obert L Chapin S. Popular weight loss strategies: A review of four weight loss techniques. Curr Gastroenterol Rep (2017) 19:61. 10.1007/s11894-017-0603-8 29124370

[112] National Library of Medicine. Expert panel report: Guidelines (2013) for the management of overweight and obesity in adults. Obesity (2014) 22:S41–S410. 10.1002/oby.20660 24227637

[113] Thaker VV. Genetic and epigenetic causes of obesity. Adolesc Med State Art Rev (2017) 28:379–405. 30416642 PMC6226269

[114] Krausz C Riera-Escamilla A. Genetics of male infertility. Nat Rev Urol (2018) 15:369–84. 10.1038/s41585-018-0003-3 29622783

[115] Xavier MJ Salas-Huetos A Oud MS Aston KI Veltman JA. Disease gene discovery in male infertility: Past, present and future. Hum Genet (2021) 140:7–19. 10.1007/s00439-020-02202-x 32638125 PMC7864819

[116] Peña-Romero AC Navas-Carrillo D Marín F Orenes-Piñero E. The future of nutrition: Nutrigenomics and nutrigenetics in obesity and cardiovascular diseases. Crit Rev Food Sci Nutr (2017) 58:3030–41. 10.1080/10408398.2017.1349731 28678615

[117] Martinez-Cordero C Kuzawa CW Sloboda DM Stewart J Simpson SJ Raubenheimer D. Testing the Protein Leverage Hypothesis in a free-living human population. Appetite (2012) 59:312–5. 10.1016/j.appet.2012.05.013 22634200

[118] Kenny DA Byrne CJ. Review: The effect of nutrition on timing of pubertal onset and subsequent fertility in the bull. Animal (2018) 12:s36–44. 10.1017/S1751731118000514 29554994

[119] Martin GB Blache D Miller DW Vercoe PE. Interactions between nutrition and reproduction in the management of the mature male ruminant. Animal (2010) 4:1214–26. 10.1017/S1751731109991674 22444618

[120] Oakberg EF. Duration of spermatogenesis in the mouse and timing of stages of the cycle of the seminiferous epithelium. Am J Anat (1956) 99:507–16. 10.1002/aja.1000990307 13402729

[121] Creasy DM. Evaluation of testicular toxicity in safety evaluation studies: the appropriate use of spermatogenic staging. Toxicol Pathol (1997) 25:119–31. 10.1177/019262339702500201 9125770

[122] Nätt D Kugelberg U Casas E Nedstrand E Zalavary S Henriksson P. Human sperm displays rapid responses to diet. PLoS Biol (2019) 17:e3000559. 10.1371/journal.pbio.3000559 31877125 PMC6932762

[123] Zegers-Hochschild F Adamson GD de Mouzon J Ishihara O Mansour R Nygren K. International committee for monitoring assisted reproductive technology (ICMART) and the world health organization (WHO) revised glossary of ART terminology, 2009. Fertil Steril (2009) 92:1520–4. 10.1016/j.fertnstert.2009.09.009 19828144

[124] Adler MI Cassidy EJ Fricke C Bonduriansky R. The lifespan-reproduction trade-off under dietary restriction is sex-specific and context-dependent. Exp Gerontol (2013) 48:539–48. 10.1016/j.exger.2013.03.007 23542072

[125] Bodin M Käll L Tydén T Stern J Drevin J Larsson M. Exploring men’s pregnancy-planning behaviour and fertility knowledge: A survey among fathers in Sweden. Upsala J Med Sci (2017) 122:127–35. 10.1080/03009734.2017.1316531 PMC5441373 28471260

[126] Shawe J Patel D Joy M Howden B Barrett G Stephenson J. Preparation for fatherhood: A survey of men’s preconception health knowledge and behaviour in England. PLoS One (2019) 14:e0213897. 10.1371/journal.pone.0213897 30893380 PMC6426231

[127] Frey KA Navarro SM Kotelchuck M Lu MC. The clinical content of preconception care: Preconception care for men. Am J Obstet Gynecol (2008) 199:S389–95. 10.1016/j.ajog.2008.10.024 19081435

[128] Jessri M Wolfinger RD Lou WY L’Abbé MR. Identification of dietary patterns associated with obesity in a nationally representative survey of Canadian adults: Application of a priori, hybrid, and simplified dietary pattern techniques. Am J Clin Nutr (2017) 105:669–84. 10.3945/ajcn.116.134684 28148504

[129] Institute of Medicine. Dietary reference intakes for energy, carbohydrate, fiber, fat, fatty acids, cholesterol, protein, and amino acids Vol. pp. . Washington, DC: The National Academies Press (2005). p. 1358.

[130] Salas-Huetos A Bulló M Salas-Salvadó J. Dietary patterns, foods and nutrients in male fertility parameters and fecundability: A systematic review of observational studies. Hum Reprod Update (2017) 23:371–89. 10.1093/humupd/dmx006 28333357

[131] Schagdarsurengin U Steger K. Epigenetics in male reproduction: Effect of paternal diet on sperm quality and offspring health. Nat Rev Urol (2016) 13:584–95. 10.1038/nrurol.2016.157 27578043

[132] Ferraretti AP Nygren K Andersen AN de Mouzon J Kupka M Calhaz-Jorge C. Trends over 15 years in ART in Europe: An analysis of 6 million cycles. Hum Reprod Open (2017) 2017:hox012. 10.1093/hropen/hox012 31486803 PMC6276702

